# Evolution of aortic pressure during normal ageing: A model-based study

**DOI:** 10.1371/journal.pone.0182173

**Published:** 2017-07-28

**Authors:** Stamatia Pagoulatou, Nikolaos Stergiopulos

**Affiliations:** Laboratory of Hemodynamics and Cardiovascular Technology, Institute of Bioengineering, Swiss Federal Institute of Technology, École Polytechnique Fédérale de Lausanne (EPFL), Lausanne, Switzerland; University of Colorado Denver School of Medicine, UNITED STATES

## Abstract

**Background:**

The age-related increase in pulse pressure (PP) and systolic blood pressure (SBP) is often attributed to alterations in the wave reflection profile and augmented contributions of the reflected waves. However, clinical evidence shows that the stiffening of the proximal aorta with age and the consequent augmentation of the forward pressure wave plays an equally important role. The relative importance of the forward and reflected wave components in essential hypertension has not yet been fully elucidated.

**Objective:**

The aim of the current investigation was to simulate the major ageing mechanisms in the arterial system and the heart using a mathematical one-dimensional model of the arterial tree and to assess the evolution of systolic and pulse pressure during normal (non-pathological) ageing.

**Methods and results:**

Our state-of-the-art 1-D model was extended to include turbulence and inertial effects of the flow exiting the left ventricle. Literature data on the age-associated changes in arterial stiffness, peripheral resistance and cardiac contractility were gathered and used as an input for the simulations. The predicted evolution of pressure and augmentation index with age followed accurately the curves obtained in a number of large-scale clinical studies. Analysis of the relative contribution of the forward and backward wave components showed that the forward wave becomes the major determinant of the increase in central and peripheral SBP and PP with advancing age.

**Conclusions:**

The 1-D model of the ageing tree and heart captures faithfully and with great accuracy the central pressure evolution with ageing. The stiffening of the proximal aorta and the resulting augmentation of the forward pressure wave is the major contributor of the systolic pressure augmentation with age.

## Introduction

Hypertension has been recognized as a major contributor to cardiovascular morbidity and mortality [1,2]. The continuous rise of systolic blood pressure (SBP) and pulse pressure (PP) over time is mainly attributed to age-associated changes in the big elastic vessels, i.e. the aorta and its major branches, which undergo gradual stiffening and dilation [3,4]. These alterations in the arterial properties and the resulting augmentation in cardiac afterload affect the cardiac structure and function, which in turn further contribute to the development of hypertension [5,6].

Even in the absence of clinical hypertension [7], age-induced arterial stiffening can be detected by an increase in the aortic Pulse Wave Velocity (PWV), a measure which serves as an independent predictor of cardiovascular and total mortality [8–10]. Central Augmentation Index (AIx), which is a composite index illustrating the augmenting effect caused by the reflected wave, has also been proposed and used extensively as a surrogate measure of arterial stiffness, although its reliability has been questioned lately [11,12]. In fact, several studies initially suggested a linear increase in both PWV and AIx with age across different populations. However, findings from Mitchell et al. [13], which were later corroborated by a number of studies [14,15], revealed a steep increase in AIx in younger individuals, followed by a slight decrease in the elderly. In this context, AIx was proposed as a rather sensitive marker of vascular ageing in younger adults [14]. The nature of AIx as well as its clinical significance are still not fully understood and require further investigation.

Another topic that has engendered controversy among researchers is the contribution of the forward and backward travelling waves on the increase of SBP with age. One possible theory proposed by O’Rourke et al. [16] is that global arterial stiffening causes the augmentation and earlier arrival of the reflected waves. This point of view was challenged by Mitchell et al. [17], who suggested that the non-uniform aortic stiffening leads to an increase in the characteristic impedance predominantly in the proximal aorta, causing an augmentation in the forward pressure wave amplitude.

The use of mathematical models of the cardiovascular system to simulate ageing could shed light to these debates and broaden our general understanding on the ageing process. Previous work from Maksuti et al. [18] has tried to do so by employing a lumped parameter (4-element Windkessel) model of the arterial tree. This simple ageing model did manage to predict systolic (SBP) and diastolic (DBP) pressure evolution quite accurately. However, the arterial tree being represented by a 4-element Windkessel model, it lacked spatial dimension and thus it was unable to capture wave transmission effects.

The objective of this study was to predict the evolution of pressure during physiological ageing using a simple heart model and a one-dimensional model of the arterial tree. We aimed in predicting faithfully not only the evolution of systolic and diastolic pressure, but also the wave characteristics, so that the forward and reflected wave components can be analyzed and other indices characterizing wave reflections (i.e., AIx) can be derived as well. Particular attention is paid on the contribution of the earlier-arriving and amplified reflection wave and the augmented forward wave with goal to assess their relative contribution in the development of isolated systolic hypertension.

## Methods

### Description of the 1-D model of the arterial tree

The ageing cardiovascular system was simulated using a detailed one-dimensional wave propagation model of the systemic arterial tree, which was first developed by Stergiopulos et al. [19] and later improved by Reymond et al. [20]. A brief overview of the *in silico* model, emphasizing on the most important modeling assumptions, is presented below. For a more thorough description, the reader is referred to the original publications [19–21].

In the 1-D model, blood pressure and flow waveforms are obtained throughout the vasculature through numerical integration of the 1-D form of the Navier-Stokes equations. Following Holenstein et al. [22], the viscoelastic properties of the arterial wall are included by a constitutive relation relating pressure and cross-sectional area. Dependency of local area compliance on pressure is described by Langewouters et al. [23] and assumed to have the same functional form in all arterial locations. Pulsatile effects on the velocity profile are modeled according to the Witzig-Womersley theory. In order to include the distal vessels of the arterial tree, 3-element Windkessel models are coupled to the terminal arterial segments. Cardiac contractility is represented by a time-varying elastance model of the left ventricle, as proposed by Sagawa [24].

The generic 1-D model was thoroughly validated by Reymond et al. [20]. A patient-specific model variant was further validated with *in vivo* measurements and was found capable of faithfully reproducing the flow and pressure waveform characteristics [21].

For the needs of our study, an improved version of this mathematical model was developed. Ventricular-vascular coupling was modified in order to include the opening of the aortic valve and the phenomena of inertia and turbulence in the flow exiting the ventricle, according to Mynard et al. [25]. Therefore, ventricular pressure, *P*_*lv*_, and proximal aortic pressure, *P*_*a*_, were no longer assumed to be equal during ejection. Instead:

$$\Delta p=P_{lv}-P_{a}=BQ|Q|+L\frac{dQ}{dt}$$

where *B* and *L* are the time-varying blood resistance and inertance, respectively.

### Physiological ageing

Previous work by Maksuti et al. [18] stressed the importance of taking into consideration changes in both the arterial network and the heart when simulating ageing. Indeed, concurrently with the gradual stiffening of the elastic vessels and the increase in peripheral resistance with age, the heart undergoes remodeling, usually under the form of ventricular wall thickening and stiffening. Cardiac changes affect cardiovascular performance and further contribute to the development of hypertension [18].

#### Arterial stiffening

Arterial stiffness is usually assessed by measurement of PWV, which is known to increase with distance from the heart. In Reymond’s model [20], PWV was approximated as a function of mean arterial lumen diameter by fitting the following inverse relation to the data reported for different arteries [26–31]:

PWV(d¯)≅ad¯b

where a = 13.3 and b = 0.3, with goodness of fit R^2^ = 0.6.

This $PWV(\bar{d})$ relation represents a normal arterial system, where arterial stiffness increases steeply as one moves from central to peripheral arteries. In a young adult, PWV is approximately 5 m/s in the elastic proximal aorta [32] and increases to approximately 10 m/s in the stiffer femoral artery [33]. It has been shown, however, that during normal ageing the elastic properties of different aortic regions do not undergo uniform alterations. In fact, with advancing age, proximal vessels lose their elasticity more markedly than the periphery does, to an extent that proximal aorta might even become stiffer than distal sites in the elderly [13,34].

Following Reymond’s concept [20], we created different empirical inverse relations between artery size and PWV for all age decades, using the local PWV increase reported by Rogers et al. [32]. Geometry–and, thus, mean arterial lumen diameter–was assumed to remain unchanged with age. Relation coefficients a and b as well as goodness of fit R^2^ are shown in Table 1 for each decade. Despite some dispersion, the relations yielded a good functional fit to literature data for all ages (R^2^>0.75), which was a significant improvement compared to the original relation.

**Table 1:** Inverse relation coefficients a and b, and goodness of fit R^2^ for each age decade.

	Age (years)					
$PWV≅\frac{a}{{\bar{d}}^{b}}$	30	40	50	60	70	80
**a**	15.48	15.59	16.33	16.68	15.91	15.29
**b**	0.502	0.458	0.447	0.428	0.372	0.345
**R**^**2**^	0.95	0.90	0.93	0.93	0.81	0.75

#### Peripheral resistance

For a given cardiac output (CO), the mean arterial pressure (MAP), i.e. the steady state component of blood pressure, is regulated solely by the peripheral arterial system. A number of studies have reported a rather insignificant decrease of CO with age [35,36], indicating that the age-induced increase in MAP is driven mainly by the increase in the resistance of the smaller vessels of the periphery. In our model, the total peripheral resistance (TPR) was calculated for each decade as the ratio of the expected MAP (see *Clinical pressure data*) to CO. The latter was assumed unchanged with age and equal to the average value reported by Katori [35], CO = 6.6 L/min. Based on the increase in the total peripheral resistance, all terminal resistances were then adapted accordingly in a uniform way.

#### Cardiac function

Age-induced changes in cardiac function and shape are still subject to intense investigation. One widely acknowledged cardiac change with age is the left ventricular wall thickening, which is present even in adults apparently free of any cardiovascular disease [37]. The reported augmentation in myocardial wall thickness occurs usually under the form of concentric hypertrophy [38]. With this compensation mechanism, the heart usually succeeds to bear the extra afterload without a significant increase in ventricular wall stress. Only under extreme conditions hypertrophy might develop to a point where wall stress becomes subnormal [39].

Surprisingly, these age-related changes in cardiac shape have been proven not to influence the overall shape of the normalized elastance curve, E_N_(t_N_) (normalized both by time and amplitude), which seems independent of loading conditions, heart rate, and contractility in both canine and human hearts [40–42]. The thickening -and thus stiffening- of left ventricular wall is reflected by an increase in the end-systolic elastance (E_es_). For the needs of our study, we assumed that ventricular wall stress is preserved with advancing age and thus E_es_ was increased in proportion to SBP, following the concept first developed by Maksuti et al. [18].

Stiffening of the ventricular wall renders ventricular filling slower, shifting the bulk of the atrial outflow later in diastole and thus gravely affecting diastolic function [6]. In our model, we assumed that end-diastolic elastance (E_ed_) was augmented proportionally to E_es_ increase.

Following the argumentation of Maksuti et al. [18], we made the assumption of an unchanged end-diastolic volume (EDV) during cardiac remodeling. In order to achieve a constant EDV, end-diastolic pressure (P_ven_) (which is almost equal to venous pressure) was increased appropriately with advancing age. Regarding heart rate (HR), studies have shown a decrease with age, hence we chose to vary HR values according to the physiological data reported by McEniery et al. [14] (average values between males and females per decade).

An overview of the ageing cardiac parameters is presented in Table 2.

**Table 2:** Overview of the ageing cardiac parameters.

	Age (years)					
Parameter	30	40	50	60	70	80
**E**_**es**_ **(x10**^**8**^ **Pa/m**^**3**^**)**	1.37	1.45	1.55	1.65	1.80	2.01
**E**_**ed**_ **(x10**^**6**^ **Pa/m**^**3**^**)**	3.43	3.63	3.67	4.13	4.50	5.03
**P**_**ven**_ **(Pa)**	861	895	937	987	1046	1131
**HR (bpm)**	73.3	72.1	70.9	69.7	68.5	67.3

E_ed_ = end-diastolic elastance; E_es_ = end-systolic elastance; HR = heart rate; P_ven_ = end-diastolic pressure.

### Clinical pressure data

Reference peripheral SBP and DBP values were obtained from the Framingham Heart Study reported by Franklin et al. [43], using the average values between normotensive groups 1 and 2. Expected central SBP was later computed using the mean pressure amplification from the proximal aorta to the brachial artery for each age decade, as documented by the Anglo-Cardiff Collaborate Trail II [44].

### Data analysis

#### 1-D model vs clinical data

The difference between the model pressure predictions and the clinical data was quantified by calculation of the normalized root-mean squared error (n-RMSE). From the model-derived pressure waveforms, the contribution of the reflected wave amplitude to the total PP was assessed by changes in augmentation pressure (AP). Subsequently, aortic pressure wave shape was assessed by calculation of the central augmentation index (AIx), i.e. the ratio of AP to central pulse pressure (PP_central_), following the methodology previously described in Murgo et al. [31].

#### 1-D model vs 0-D model

Aortic pressure waves were separated into their forward and backward wave components, following the standard analysis in frequency domain [45]. Characteristic impedance at the root of the ascending aorta was estimated by averaging the input impedance modulus (Zc) in the frequency range of 3–15 Hz. Forward wave pulse pressure (PP_forward_) was defined as the difference between the systolic and diastolic pressure of the forward wave component. Wave separation analysis was applied to the 0-D model of Maksuti et al. [18], as well as to the 1-D model and the ratios of forward wave PP to central PP at the ascending aorta were plotted as a function of age.

## Results

The values of the simulation-derived hemodynamic parameters are presented in Table 3, grouped by decade of age.

**Table 3:** Simulation derived hemodynamic parameters according to age category.

	Age (years)					
Parameter	30	40	50	60	70	80
Brachial SBP (mmHg)	109.7	114.0	121.9	129.0	136.8	141.3
aortic SBP (mmHg)	97.7	105.1	112.1	117.8	126.2	129.8
MAP (mmHg)	84.8	88.8	91.6	94.8	95.8	95.4
DBP (mmHg)	73.9	77.0	77.0	77.9	74.1	73.3
PP_peripheral_ (mmHg)	37.4	37.7	45.5	51.3	61.4	68.8
PP_central_ (mmHg)	23.9	28.1	35.2	39.9	52.1	56.5
PP ampl (ratio)	1.57	1.34	1.29	1.28	1.18	1.21
AP (mmHg)	0.6	2.4	6.1	7.2	6.3	7.1
AIx (%)	2.7	8.6	17.0	18.1	12.5	12.5
Inflection Point (ms)	154	136	123	114	124	120

AIx = augmentation index; AP = augmentation pressure; DBP = diastolic blood pressure; MAP = mean arterial pressure; PP = pulse pressure; PP ampl = pulse pressure amplification; SBP = systolic blood pressure.

**Fig 1:**
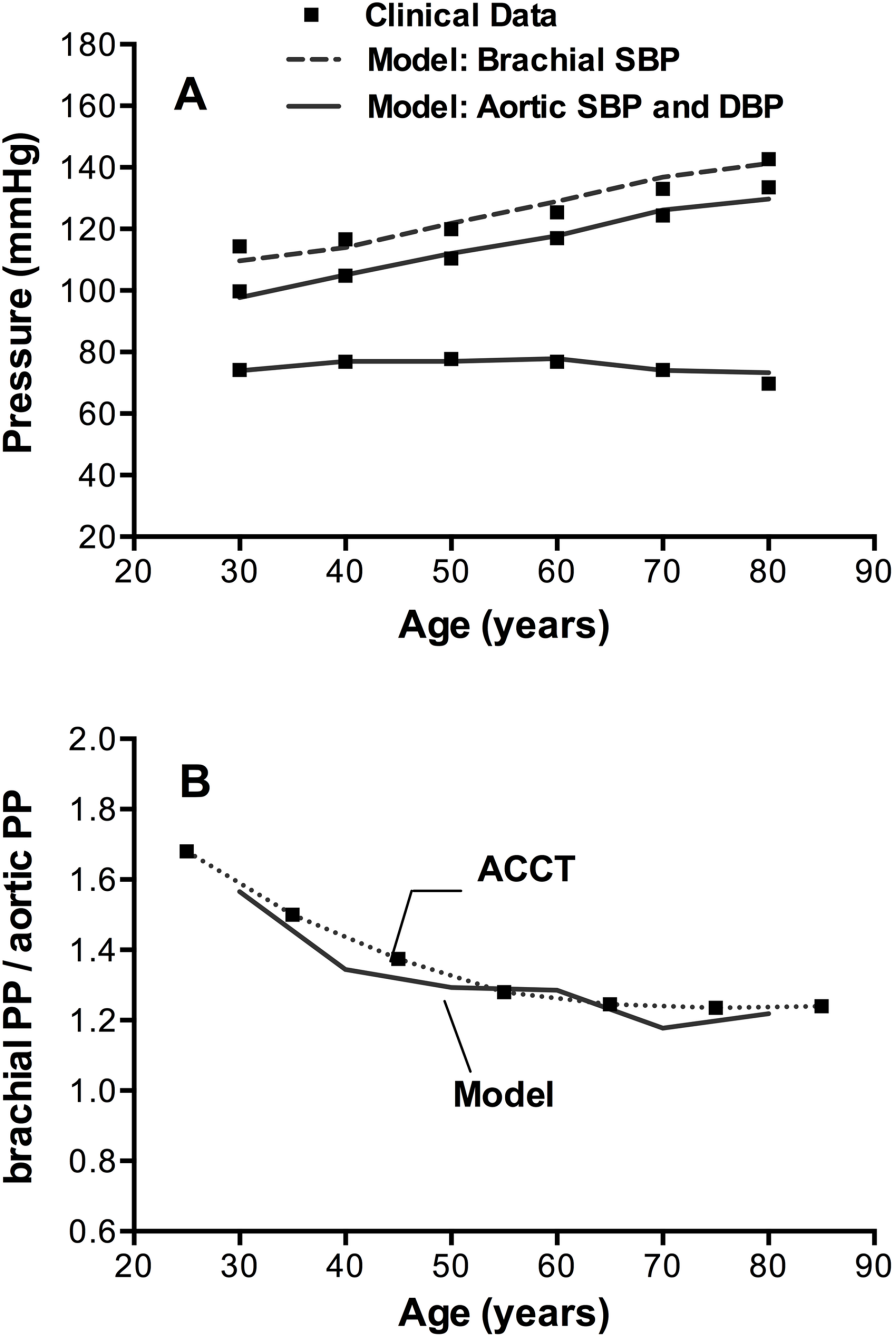
Model pressure predictions against clinical data for the time frame of 30 to 80 years of age. *Panel A*. Model-derived brachial SBP (dashed line) and aortic SBP and DBP (solid line) against the reference data [43,44] (squares). *Panel B*. Model-derived ratio of peripheral (brachial) PP to central (aortic) PP (solid line) against the measurements from ACCT trial [44] (squares-dotted line).

The evolution of brachial SBP and aortic SBP and DBP with age is depicted also in Fig 1A in comparison to the clinical pressure data described above. As expected, the model predicted that both central and peripheral SBP increased progressively over time. Aortic SBP increased from 98 mmHg at 30 years to 130 mmHg at 80 years of age, while brachial SBP increased from 110 mmHg to 141 mmHg during the same time period. In contrast, DBP initially increased until 50 years and then followed the well anticipated decline. This resulted in the expected widening of both central and peripheral PP with age. Goodness of fit parameter n-RMSE was calculated and found equal to 2.2%.

Central SBP increased more with age than peripheral SBP did, resulting in a loss of PP amplification (defined as the ratio of brachial PP to aortic PP). As depicted in Fig 1B, the ratio varied from 1.57 at 30 years to less than 1.22 at 80 years of age and was in close agreement with the data from the Anglo-Cardiff Collaborate Trial II (ACCT) [44]. Note that the decrease in PP augmentation was steeper for young adults, conforming to the ACCT findings.

**Fig 2:**
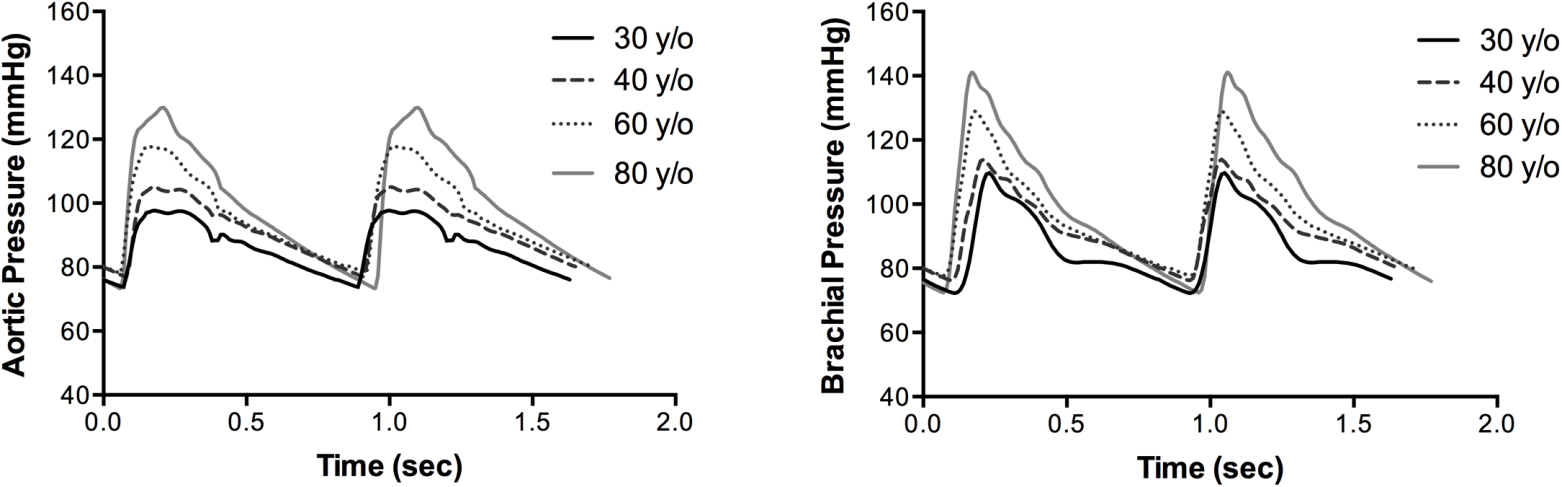
Pressure waveforms during ageing. Aortic (*left*) and brachial (*right*) pressure waveforms for the age decades: 30 years (black solid line), 40 years (dashed line), 60 years (dotted line), 80 years (grey solid line).

Fig 2 shows predicted pressure waveforms at the ascending aorta and the brachial artery and their evolution from 30 to 80 years of age. As expected, the pressure pulse wave is shown to arrive earlier in the brachial artery with advancing age due to the higher PWV.

**Fig 3:**
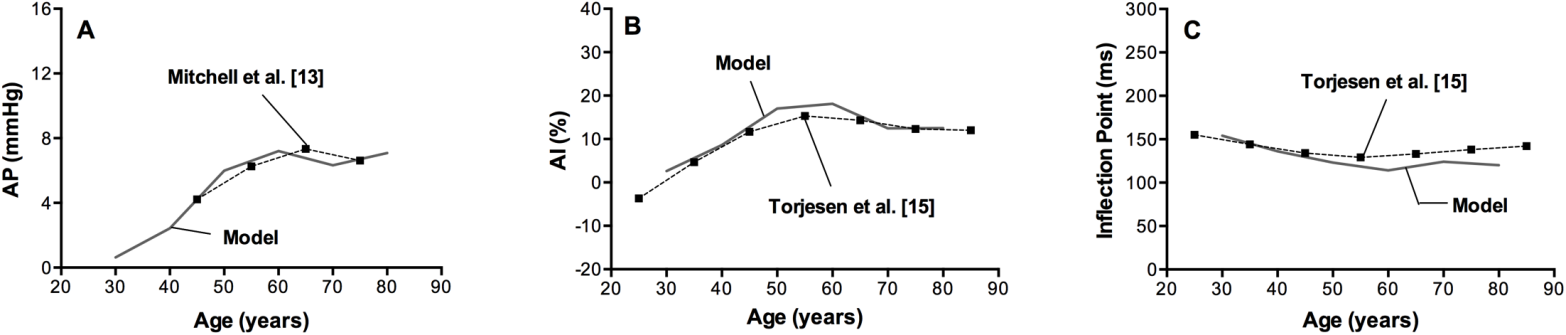
Model predictions (solid line) against clinical data from Mitchell et al. [13] and Torjesen et al. [15] (black squares). *Panel A*. Augmentation Pressure, *Panel B*. Augmentation index, *Panel C*. Inflection Point.

Augmentation pressure (AP) is plotted in Fig 3A against the reference AP values [13]. As previously suggested by Mitchell et al. [13], we found that AP changed minimally with advancing age. Aortic augmentation index as well as inflection point were also computed for each age decade. In our model AIx was found to increase steeply in young adults and actually decline after 60 years of age. Our results were highly consistent with the findings of Torjesen et al. [15] (average values between males and females), as shown in Fig 3B and 3C.

**Fig 4:**
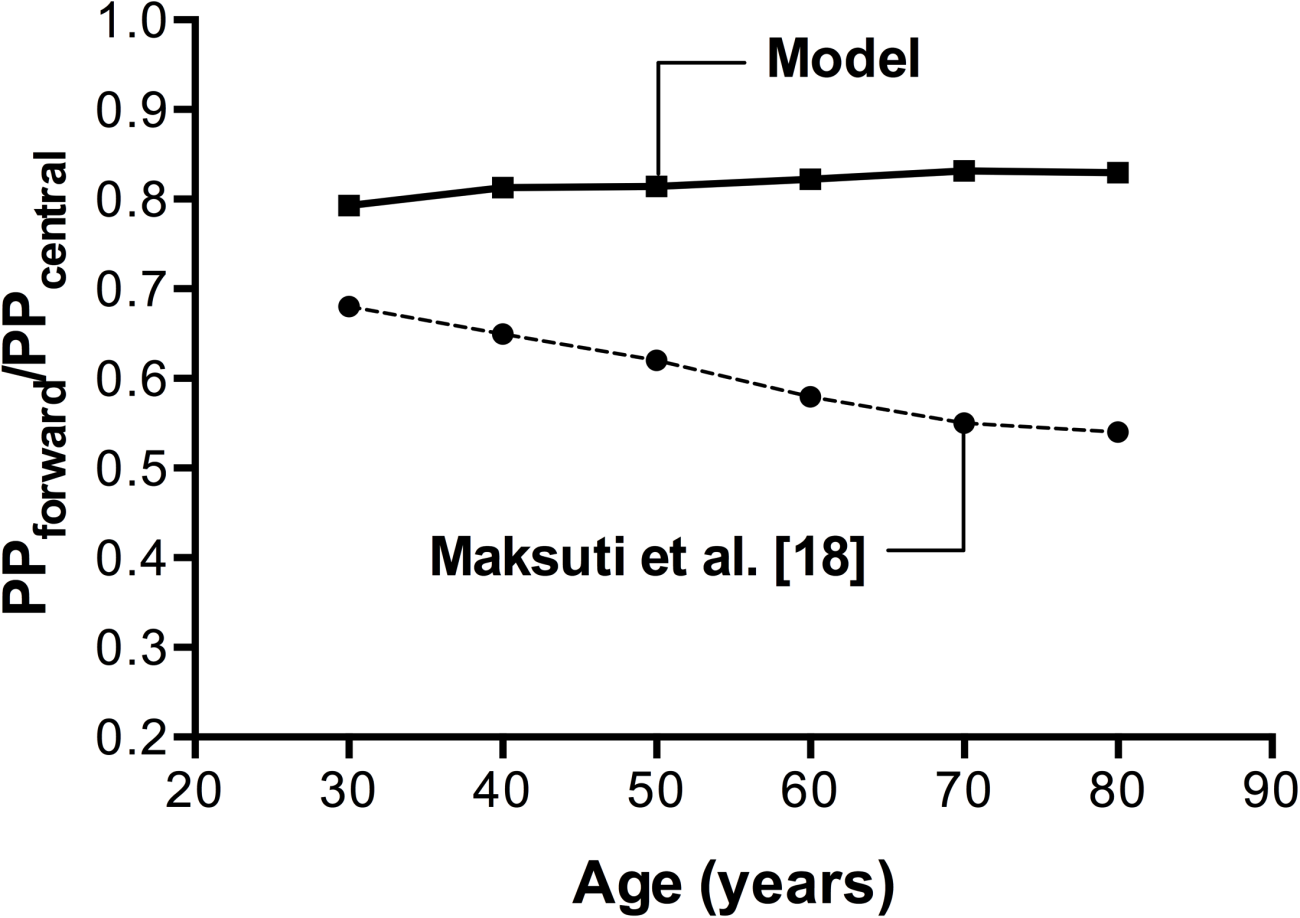
Contribution of forward pressure wave to pressure augmentation during ageing, as assessed by the PP_forward_/PP_central_ ratio. 1-D model results (squares—solid line) compared to the 0-D model results [18] (dots–dashed line).

Forward wave pulse pressure (PPforward), computed after the wave separation analysis, was used in order to assess the contribution of the forward wave to systolic pressure augmentation during ageing. The ratio of the forward wave pulse pressure to the central pulse pressure (PPforward/PPcentral) is presented in Fig 4, compared to the results obtained by the 0-D model of Maksuti et al. [18]. In our model, the ratio increased from 0.78 to 0.83 between the ages of 30 to 80 years old, while the 0-D model predicted a decline.

## Discussion

The present study aimed to achieve three goals. First, to simulate the evolution of arterial pressures during the physiological ageing process using a realistic 1-D model of the systemic arterial tree. The original 1-D model, upon which our work was based, was developed in our laboratory [19,20] and is considered to be one of the most complete and thoroughly validated models available in literature. This powerful computational tool has successfully provided a solid foundation for a number of subsequent studies [46–49].

In order to gain in-depth insight into the ageing cardiovascular system, we decided to modify the original version of the 1-D model, which previously assumed left ventricular and aortic pressure to be equal during ejection. This assumption is limiting since there are flow phenomena created in the aortic valve that lead to differences between the ventricular and aortic pressure waveforms both in magnitude and phase [25]. An elevated pressure difference might even be indicative of aortic stenosis. Although in our work no pathological cases were simulated, omitting inertial effects and turbulence losses at the aortic valve, a key component of the ventricular-vascular coupling, would reduce the accuracy of the aortic wave shape even in physiological conditions. Indeed, after including in our simulations the inertia and turbulence model proposed by Mynard et al. [25], AIx predictions were significantly improved, with the n-RMSE decreasing almost threefold.

Based on data from a wide range of literature, we expanded the generic 1-D model—representative of a young healthy male—into a model of the arterial tree adaptable to all adult ages. A key point in simulating arterial ageing was to describe in a mathematical way the progressive and heterogeneous stiffening of the arterial network, which has been widely recognized as a determinant for the rise in SBP. To achieve this, we first examined closely the inverse relation between artery size and PWV that was originally proposed by Reymond et al. [20]. This relation was created combining reference data on local aortic and peripheral PWV measurements from different populations and exhibited a relatively poor correlation coefficient of R^2^ = 0.6. What was not taken into consideration by Reymond et al. [20], however, was the fact that the aforementioned populations were of different age groups, some even exceeding 55 years old. Given the high dependency of PWV on age, this contributed to the significant dispersion observed between the average “fitted” curve and the literature data. Indeed, after the age factor was accounted for, the age-dependent $PWV(\bar{d})$ relations fitted well to the literature data and the dispersion decreased for all age decades (R^2^>0.75 in all cases).

The second goal was to validate the predictions of the ageing model via a comparison to published data from large-scale clinical studies. Despite the simplicity of the rules we applied, and despite the fact that we did not take into consideration how the arterial geometry evolves with time, our simulations were surprisingly accurate; our prediction of SBP and DBP reproduced the pattern reported by the Framingham heart study [43] and the Anglo-Cardiff Collaborate Trial II [44]. We achieved an n-RMSE of 2.2%, whereas the 0-D model from Maksuti et al. [18] achieved a n-RMSE of 5.1% when compared to the same clinical data. In addition, our *in silico* ageing model provided pressure waveforms that, from a qualitative point of view, captured faithfully the actual wave characteristics at the ascending aorta and the brachial artery.

Furthermore, we evaluated the change with age of the brachial to aortic PP ratio and found that our model was able to follow the anticipated evolution: a marked decrease in young ages followed by a less marked change in senescence. This decrease in PP augmentation is a key feature of ageing and has become the subject of intense investigation, since it serves as a sign of adverse clinical outcomes [50]. Mitchell et al. [13] interpreted this decrease as the combination of changes in both the wave reflection and arterial stiffness. He suggested that in young adults, as arterial stiffness increases steeply from the proximal aorta towards the distal branches, local reflections occur relatively earlier in time, leading to a greater PP augmentation. With advancing age, the significant increase in proximal PWV reduces or might even reverse the arterial stiffness gradient, shifting the reflection sites distally and apparently reducing amplification.

According to Avolio et al. [51], the factor most closely associated with PP amplification is AIx. In prior studies, AIx has been extensively used as a surrogate measure of arterial stiffness, although it seems to be a rather composite measure that synthesizes the impact of different parameters, i.e. arterial stiffness, heart rate, and reflection wave properties. In contrast to findings from Hayward et al. [52], our model results revealed a clearly non-linear relationship between age and AIx. In young individuals, AIx was found to rise steeply with age in contrast to older individuals, whose AIx decreased minimally. This pattern has been previously reported by a plethora of studies [13–15]. In this context, we agree that AIx might serve as a rather sensitive measure of vascular ageing in younger adults, as suggested by McEniery et al. [14].

These outcomes, which are clearly associated with non-uniform alterations in the wave reflection profile, inspired us to further investigate the relative changes in the aortic forward and backward pressure waveforms during ageing. Therefore, we set as third goal of our study to quantify and assess the contribution of each wave component to the development of essential hypertension. Absolute values of forward wave amplitude were found to increase with age, whereas AP (which serves as an index of the reflected wave amplitude) changed only minimally. The relative contribution of the wave components was assessed by the ratio of the forward wave PP to central PP, which increased with advancing age. Hence, our findings supported the theory proposed by Mitchell et al. [13,17], according to which it is the forward wave that becomes the major determinant of the increase in central and peripheral SBP and PP with advancing age. This finding is contradicting the predictions of the 0-D model [18], which failed to capture the wave transmission effects.

### Limitations and future work

Limitations of the 1-D model with respect to the formulation of the governing equations are acknowledged and discussed in detail in the original publication by Reymond et al. [20].

Cardiac function was adapted for each decade according to the physiological rules employed by Maksuti et al. [18]. The shape of the normalized elastance function E_N_(t_N_) (in time and magnitude), is suggested to be invariable among hearts, particularly during early contraction [40–42]. In fact, the isovolumic relaxation phase of the cardiac cycle is prolonged in older individuals, due to the slower ventricular filling [6]. The magnitude of the alteration in the timing of the relaxation and contraction phase is, however, rather negligible in healthy adults.

Central arterial geometry is known to change significantly during normal ageing [53–55]. To adapt to the changes in hemodynamic stresses acting on the arterial wall, the proximal aorta thickens, lengthens, enlarges in diameter and becomes tortuous in older individuals, as described in [55]. The same study has demonstrated a close relationship between these geometrical alterations in proximal aorta and the increase in central and brachial SBP. This seems reasonable given that an alteration in regional aortic diameter and length would clearly affect PWV and pulse transit time. Whether arterial diameter changes themselves are an important pathophysiological factor remains unknown. In an attempt to address this question, we assessed the effects of aortic geometric changes in model predictions, using the *in vivo* data provided by Redheuil et al. [55] on the evolution of the ascending, proximal and distal aortic diameters with age. Surprisingly, central systolic and diastolic pressure values were minimally affected (n-RMSE changed by less than 3%), suggesting that geometric changes in the aorta alone are not the determining factor in the alteration of central aortic hemodynamics with age. Nevertheless, more literature data on the aforementioned geometric changes will enable us to include in our simulations more precise models of the arterial remodeling and study in depth its significance.

Furthermore, gender-related differences in cardiovascular ageing are important, as supported by a substantial volume of literature. The increase in central SBP and PP with age is less prominent in men than women [14]. This variation is often correlated with the fact that large artery stiffness and pulsatility is on average more pronounced in older females [3]. Prior studies have documented significant wave reflection differences between the two sexes, with women systematically demonstrating higher AIx values and larger reflection waves than men [13]. This characteristic is often attributed to their shorter height and the closer physical proximity between the heart and the reflection sites, although this explication might not be sufficient [56]. Moreover, given that women have a significantly longer life span and a lower risk of cardiovascular disease, it is likely that cardiac structure is better preserved in the feminine heart [57]. All these observations possibly reflect intrinsic differences in the cardiac and arterial properties between the two genders. However, gender-related variations in cardiovascular anatomy and physiology were not accounted for in the present study due to paucity of clinical data and thus the present work is most pertinent for the evolution of pressure in males.

Future work will focus on generating more data in order to allow for a quantitative validation of the ageing model on a patient-specific and gender-specific approach, where the generic 1-D model will be tuned according to the measured cardiovascular geometry and properties of each patient. This way, the model-derived pressure and flow waveforms will be quantitatively and qualitatively compared with the measured ones.

## Conclusion

We developed a 1-D model of the ageing cardiovascular system, which can serve as a tool for investigating how cardiac and arterial changes influence hemodynamic characteristics. The model output has been found highly consistent with published data from large-scale studies, particularly in terms of systolic and diastolic pressures, wave shape and reflection indices. Examination of the model-derived wave reflection profile revealed a pronounced correlation between the augmentation in the forward wave amplitude and the increase in SBP and PP over time.

This study lays the ground for further investigation of the ageing mechanisms which contribute to the development of essential hypertension and lead to cardiovascular disease. New detailed information about age-related changes in aortic geometry and properties would help further improve the accuracy of the mathematical models of the ageing cardiovascular tree.
